# Supplementary material for: Ultrasmall ATP-Coated Gold Nanoparticles Specifically Bind to Non-Hybridized Regions in DNA
Source: Nanomaterials (Basel). 2023 Dec 5;13(24):3080. doi: 10.3390/nano13243080 (PMC10745773; doi:10.3390/nano13243080)
Supplement: Supplementary file 1 [file nanomaterials-13-03080-s001.zip › nanomaterials-2737232-supplementary.pdf]

Ultrasmall ATP-Coated Gold Nanoparticles Specifically Bind to Non-Hybridized Regions in DNA

Liat Katrivas, Asaf Ben-Menachem, Saloni Gupta and Alexander B. Kotlyar *

Department of Biochemistry and Molecular Biology, George S. Wise Faculty of Life Sciences and
Nanotechnology Center, Tel Aviv University, Ramat Aviv, Tel Aviv 69978, Israel; liatkatrivas@mail.tau.ac.il (L.K.); asafb2@mail.tau.ac.il (A.B.-M.); salonigupta@mail.tau.ac.il (S.G.)

* Correspondence: s2shak@tauex.tau.ac.il

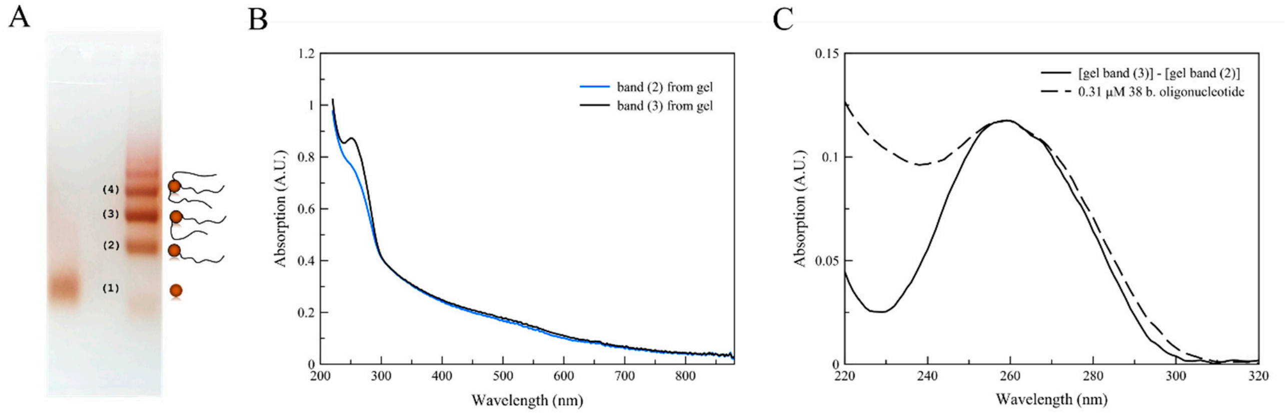

Figure S1. Electrophoretic separation and absorption spectroscopy analysis of ATP-NP-DNA conjugates bearing discrete number of DNA strands per particle. ATP-NPs were synthesized as described in Materials and methods. The particles were incubated with 2.5 molar excess of 38-base ssDNA containing thiol residues at the 5′-end of the DNA strand for 16 hours (see Materials and methods for incubation conditions). The incubation was then concentrated using 10 KDa 0.5 mL Amicon Ultra centrifugal filters to a final volume of ~30 μL. **A** - The incubation (right lane in panel A) as well as ATP-NPs (left lane in panel A) were loaded onto a 5% agarose gel and electrophoresed at 100 V in an ice cold-water bath for about 1 hour. Schematic drawing of conjugates corresponding to the coloured bands (marked as 1,2,3 and 4) is shown on the right of the gel image. The coloured areas "2" and "3" were cut from the gel with a razor blade, placed into dialysis bags filled TAE buffer and electroeluted from the gel. **B** - Absorption spectra of the electroeluted conjugates. Blue and black curves correspond to the conjugates electroeluted from the gel areas marked as "2" and "3" respectively. The spectra have been normalized to unity absorbance at 420 nm. **C** - Differential spectra of the conjugates electroeluted from areas: 3 and 2 (black curve). Dashed curve – the spectrum of 0.31 μM 38-base ssDNA (used for conjugation with the particles).

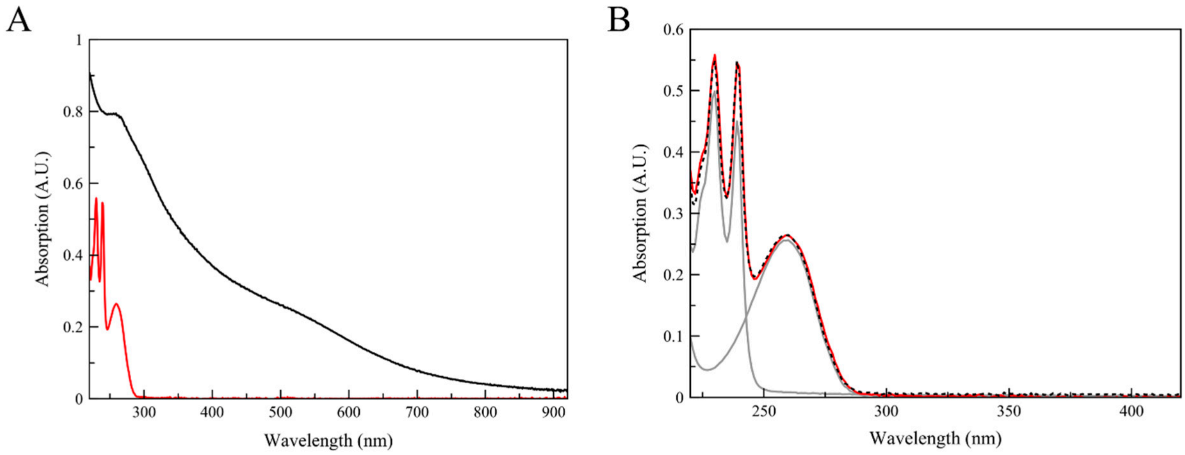

Figure S2. Absorption spectroscopy of ATP-NPs. The particles were prepared as described in Materials and methods. A – Absorption spectra of 0.43 μM ATP-NPs (black curve) treated for 5 minutes with 10 mM KCN (red curve). B – Simulation of the CN-treated spectrum of the particles (red curves on A and B). Black dotted curve correspond to the sum of 16.9 μM ATP and 107.7 μM $Au(CN)_2^-$ (grey curves).

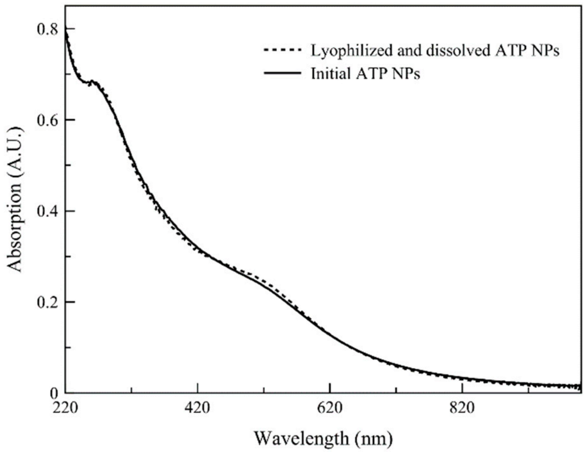

Figure S3. Absorption spectroscopy of ATP-NPs (continuous curve) and the lyophilized particles (dashed curve). The particles were prepared as described in Materials and methods. ATP-NPs were lyophilized and dissolved in small volume of water. The spectra were normalized to unity absorbance at 420 nm.

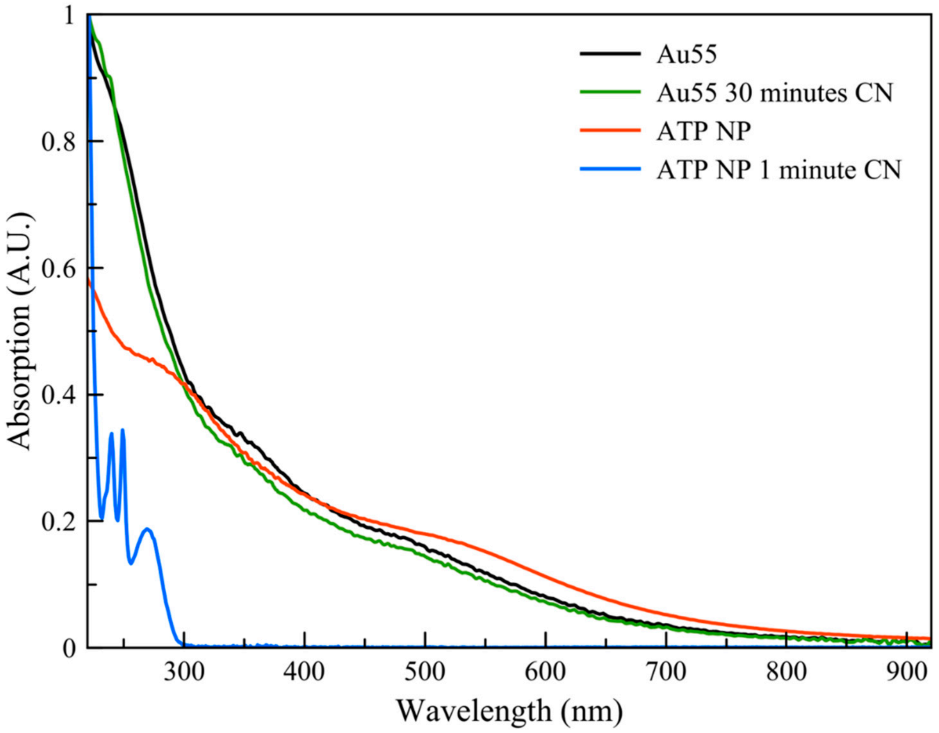

Figure S4. Absorption spectra of: ATP-NPs (red curve) treated for 1 min with 10 mM KCN (blue curve) and Au_{55}-NPs (black curve) treated for 30 min with 10 mM KCN (green curve). The appearance of sharp peaks with absorption maxima at 240 and 230 nm is clearly seen in the spectrum of KCN-treated ATP-NPs (blue curve); the spectrum of KCN-treated Au_{55}-NPs (green curve) is quite similar to that of the untreated particles (black curve).

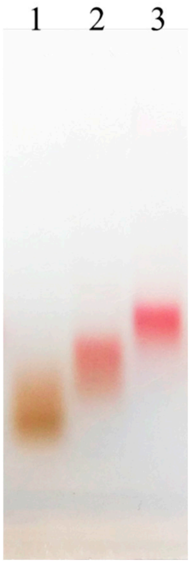

Figure S5. Electrophoresis of 1.4 nm commercial gold nanoparticles (Nanoprobes Inc., USA; catalogue #2023) incubated for 1 min with 1 mM ascorbate and: 0.2 (lane 2) or 1 mM (lane 3) $HAuCl_4$ (see Materials and methods for details). The particles were concentrated by ultrafiltration and electrophoresed on 4% agarose gel at 100V in TAE buffer at 15-20°C.

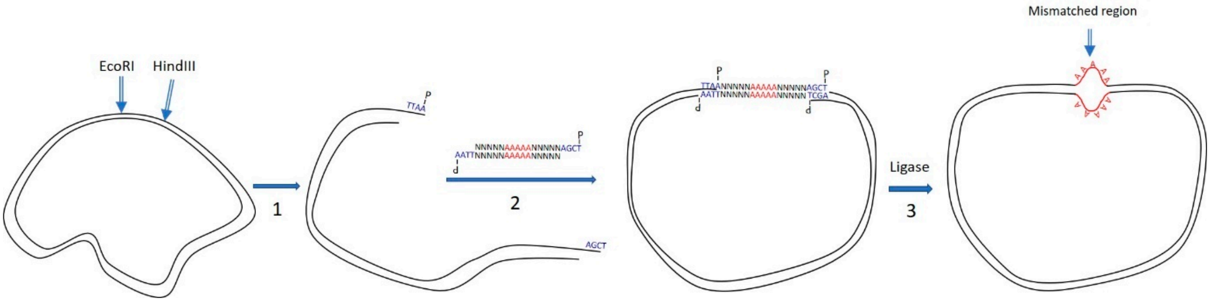

Figure S6. Schematic drawing of 5A/5A-fragment insertion into pUC19. 1- pUC19 is cleaved by two sequence-specific endonucleases, EcoRI and HindIII. The product of the enzymatic cleavage is a linear plasmid comprising 4-nucleotide overhangs at 5′-ends of each of DNA strands. 2– Insertion of a short dsDNA construct containing a middle 5A/5A fragment (red letters), two 15 bp hybridized fragments on both sides of the 5A/5A (showed by letters “N”; each N corresponds to 3 nucleotides in the sequence) and 4-nucleotide sequences complementary to the overhang sequences of the cleaved plasmid at the 5′-end of each strand (blue letters). Both strands composing the construct are phosphorylated (letters “P”) at the 5′-ends. 3- Enzymatic (catalysed by DNA ligase) ligation of the construct into the plasmid.

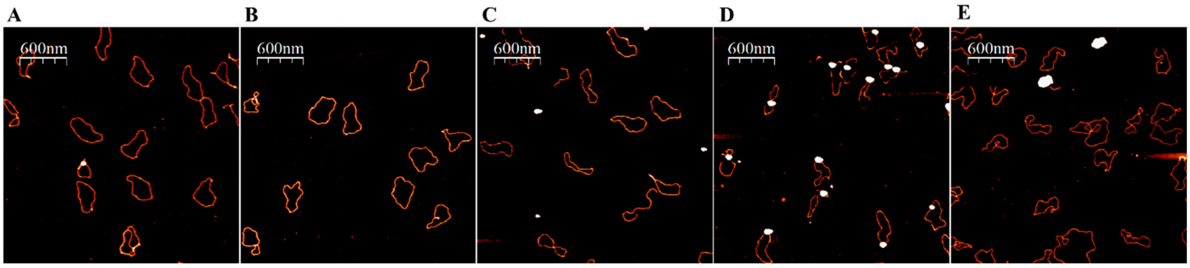

Figure S7. Conjugation of pUC(5A/5A) with: A - 5 nm (in diameter) citrate-protected GNPs; B – 1.4 nm (in diameter) commercial Au_{55}-NPs; C - ATP-NPs treated with BSPP; D - ATP-NPs. E – Conjugation of the plasmid lacking 5A/5A region with ATP-NPs. The plasmid, pUC(5A/5A), was prepared as illustrated in Figure S6 and described in Materials and methods. pUC(5A/5A) was conjugated with ATP-NPs and separated from the excess of the particles as described in legend to Figure 3. Deposition of the conjugate and enlargement of the particle associated with the plasmid was also conducted as in Figure 3.
